# Analysis of micrognathia with obstructive sleep apnea syndrome improved by a combination of Le Fort I with horseshoe osteotomies, mandibular distraction osteogenesis, and genioplasty

**DOI:** 10.1002/ccr3.6479

**Published:** 2022-12-08

**Authors:** Shinya Koshinuma, Takafumi Fujii, Takeshi Okamura, Yasuyuki Asada, Yoshisato Machida, Gaku Yamamoto

**Affiliations:** ^1^ Department of Oral and Maxillofacial Surgery Shiga University of Medical Science Otsu Japan; ^2^ Department of Oral and Maxillofacial Surgery Toyosato Hospital Inukami Japan; ^3^ Department of Oral and Maxillofacial Surgery Nagahama Japanese Red Cross Hospital Nagahama Japan

**Keywords:** jaw deformity, microgenia, obstructive sleep apnea syndrome, orthognathic surgery

## Abstract

The relationship between microgenia and obstructive sleep apnea syndrome is well known. 27‐year‐old woman. She underwent a combination of Le Fort I with horseshoe osteotomies and mandibular distraction osteogenesis and genioplasty. She was satisfied with the aesthetics of her face, with an AHI of 7.8/h.

## Graphical abstract

We discuss about Le Fort I with horseshoe osteotomies (LF + HS) and mandibular distraction osteotomy for patients with small mandibular disorders with OSA and bilateral mandibular condyle resorption.

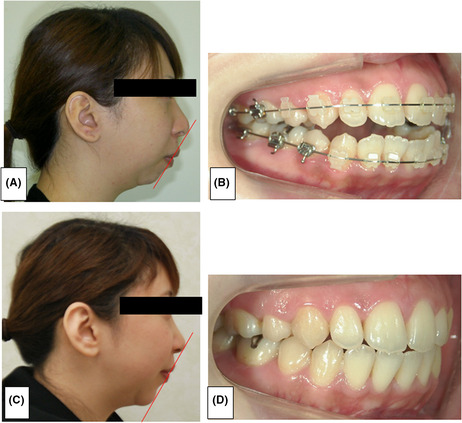

## 1 INTRODUCTION

Obstructive sleep apnea syndrome (OSA) is a disease in which apnea appears due to obstruction of the pharynx during sleep. Several reports have described that abnormal maxillofacial morphology such as small mandibular disorder is involved in the pathophysiology of OSA.^1^, ^2^ Furthermore, the influence of maxillofacial morphology is large in Asian races wherein the incidence of OSA due to obesity is low.^3^

Here, we discuss about Le Fort I with horseshoe osteotomies (LF + HS) and mandibular distraction osteotomy with horseshoe osteotomy for patients with small mandibular disorders with OSA and bilateral mandibular condyle resorption. After performing surgery (mandibular distraction osteogenesis, MDO) and then genioplasty (GP), we encountered a case in which OSA could be improved in addition to small mandibular disease and malocclusion.

## 2 MATERIAL AND METHOD

Patient: A 27‐year‐old woman.

First visit: March 13, 2012.

Primary complaint: Hoping to get treated for micrognathia and malocclusion.

Family history and medical history: No special notes.

Current medical history: On March 13, 2012, she visited our hospital with a primary complaint of micrognathia and malocclusion. We referred to an orthodontist for the diagnosis of her jaw deformity and preoperative orthodontic treatment. When she returned to our department on March 10, 2013, after the completion of the preoperative orthodontic treatment, she complained of drowsiness during daytime. Therefore, we referred her to the sleep outpatient department, where the Epworth Sleepiness Scale (ESS) score was recorded at 14 points and the apnea–hypopnea index (AHI) was 22.5/h, based on which moderate OSA was diagnosed.

Present illness: Systemic findings: Nutritional status was good, height was 163.6 cm, body weight was 61.8 kg, and body mass index was 23.0 kg/m^2^, indicating the absence of obesity according to the criteria of the Japan Society for the Study of Obesity.^4^

**FIGURE 1:**
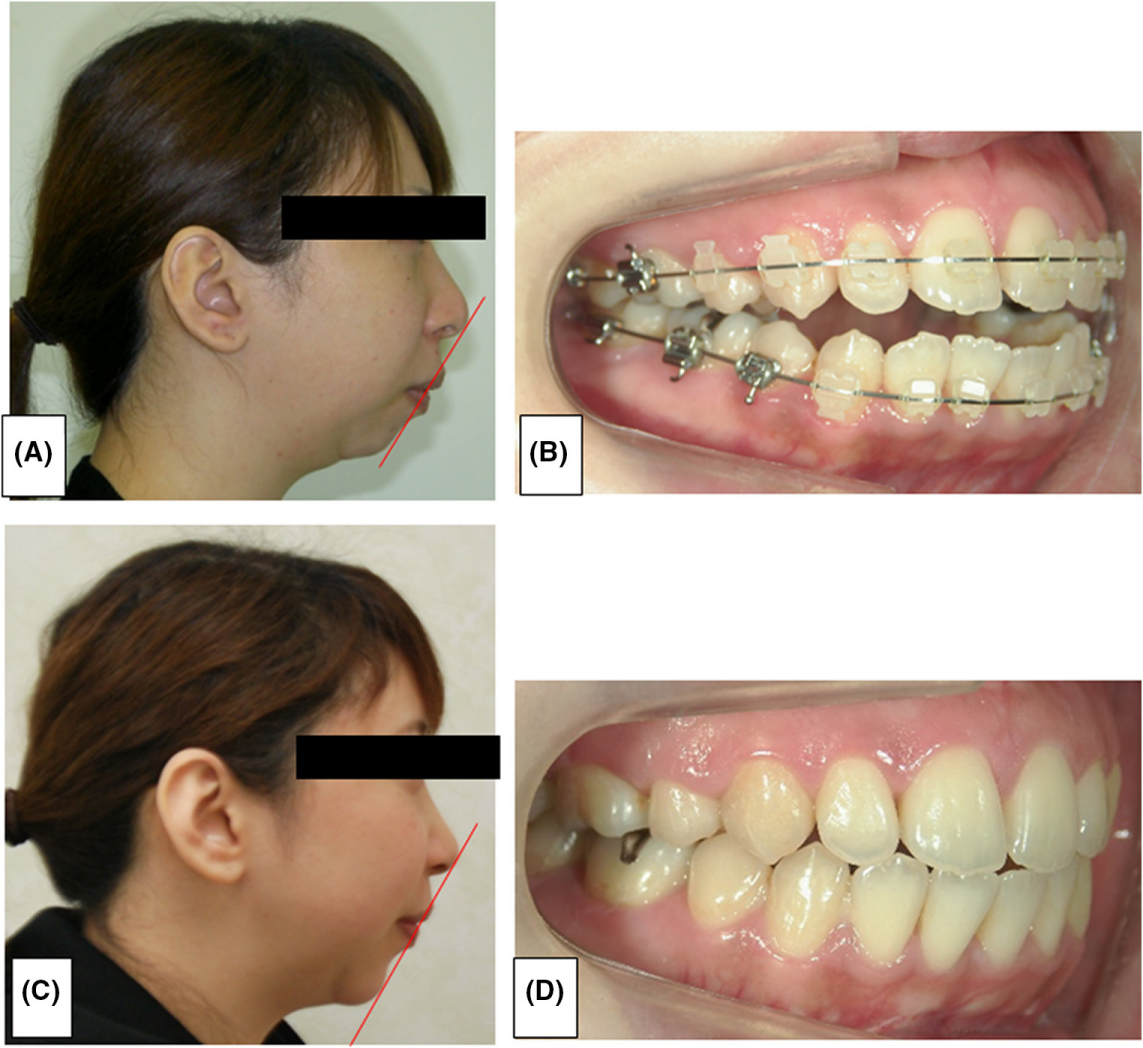
(A): Preoperative side view photograph (March 7, 2013). (B): Preoperative intraoral photograph (March 7, 2013). (C): Profile photograph at the end of our hospital examination 1 year and 3 months after GP (February 17, 2015). (D): Intraoral photograph at the end of our hospital examination 1 year and 3 months after GP (February 17, 2015).

Extraoral findings: Her face was symmetrical and long, the lateral side of the face was convex with marked mandibular retraction, and the chin was markedly retreated (Figure 1A). No clicking sound or pain was found in the temporomandibular joint.

Intraoral findings: The occlusal state revealed angle II grade on both sides, the horizontal overlap was +3.0 mm, and the vertical overlap was −2.0 mm (Figure 1B). The exposed gingiva of the maxillary anterior teeth measured 5 mm.

No abnormal findings were detected in the size or morphology of dentition and tongue, and no hypertrophy was observed in the uvula or palatine tonsils.

**FIGURE 2:**
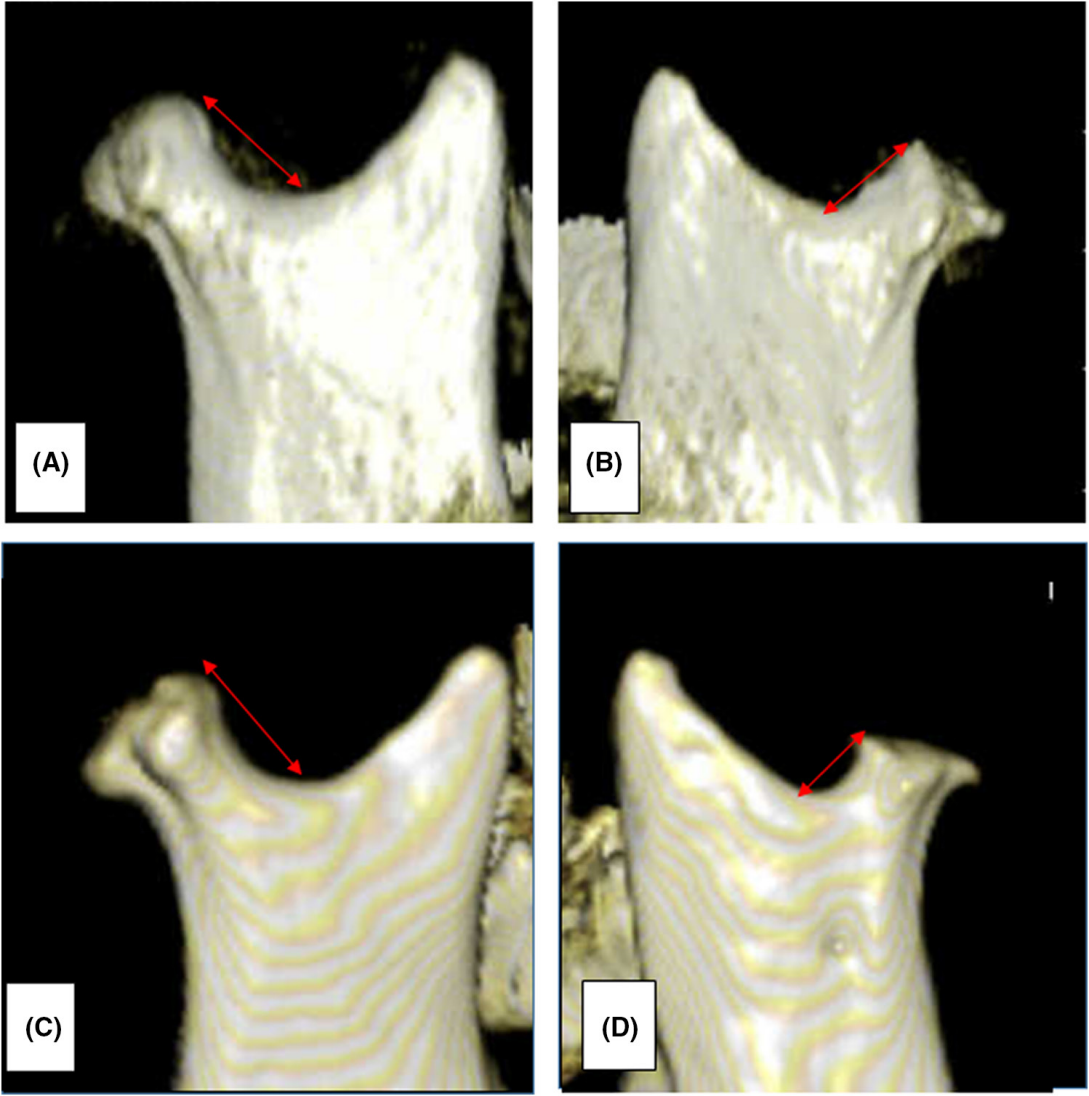
3D‐CT image of the mandibular condyle. (A): Preoperative right mandibular condyle (March 7, 2013) (B): Preoperative left mandibular condyle (March 7, 2013) (C): Right mandibular condyle 1 year and 3 months after LF + HS and MDO and 11 months after GP (August 14, 2014). (D): Left mandibular condyle 1 year and 3 months after LF + HS and MDO and 11 months after GP (August 14, 2014).

Imaging findings (March 14, 2013): Three‐dimensional computed tomography (3D‐CT) images revealed absorption images on both mandibular condyles. (Figure 2A,B).

**FIGURE 3:**
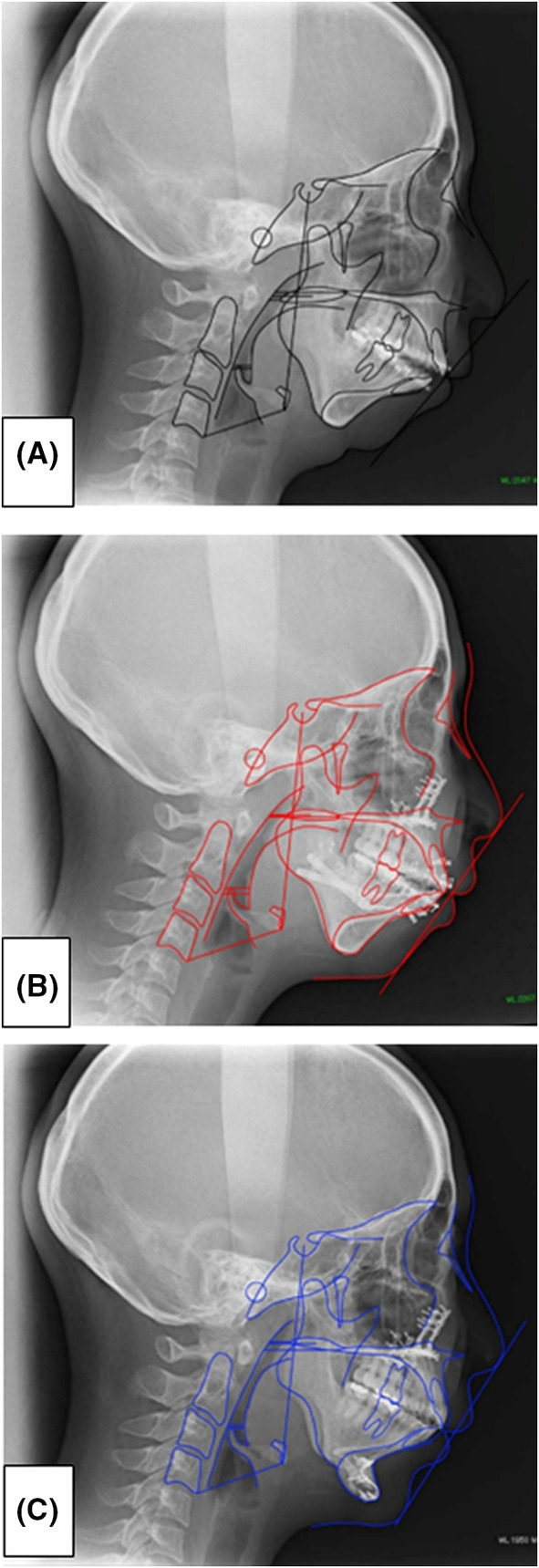
Cephalogram. (A): Black line: Preoperative (March 7, 2013). (B): Red line: LF + HS, 7 months after MDO (December 13, 2013). (C): Blue line: LF + HS, 2 years and 6 months after MDO, 1 year and 11 months after GP at the end of the examination (November 17, 2015).

In the cephalometric analysis, SNA (81°) was within the reference value, and SNB (69°) was smaller than the reference value. The ANB (12°) was larger than the reference value, the facial angle (68°) was smaller than the reference value, the maxilla was within the reference value, and the mandible was in the posterior orientation (Tables 1 and 2, Figure 3A).

**TABLE 1:** Values of ESS and AHI, before surgery (April 18, 2013), 5 months after LF + HS and MDO (October 10, 2013), 9 months after GP (September 1, 2014)

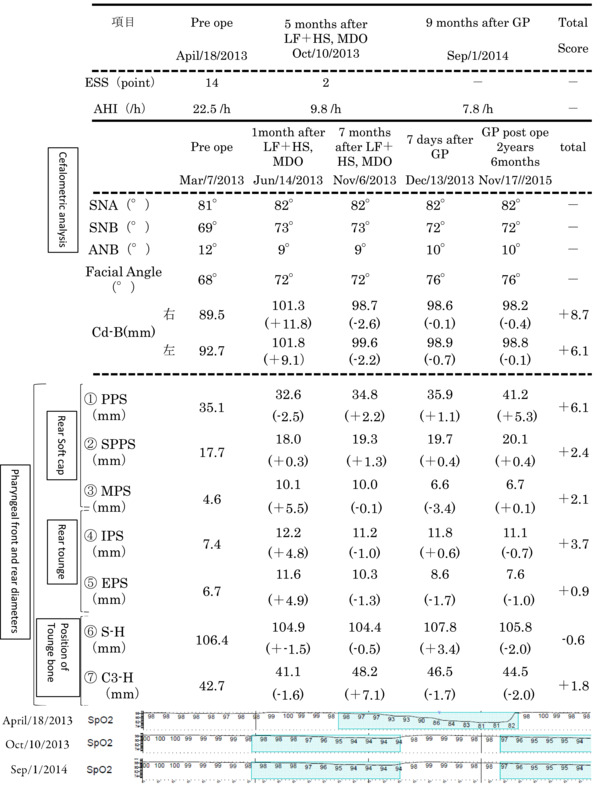

*Note*: Cephalometric analysis results (SNA, SNB, ANB, facial angle, Cd–B point distance, and measurement results of anterior–posterior pharyngeal diameter and tongue bone position), before surgery (March 7, 2013), immediately after LF + HS and MDO surgery (June 14, 2013), 7 months after LF + HS and MDO surgery (November 6, 2013), immediately after GP surgery (December 13, 2013), 2 years and 6 months after LF + HS and MDO, 1 year and 11 months after GP (November 17, 2015). The figures in parentheses are the difference between June 14, 2013 (1 month after LF + HS, MDO) and March 7, 2013 (preoperative), and the difference between November 6, 2013 (7 months after LF + HS, MDO) and June 14, 2013 (1 month after LF + HS, MDO), the difference between December 13, 2013 (7 months after LF + HS, MDO, immediately after GP surgery) and November 6, 2013 (6 months after LF + HS, MDO), the difference between November 6, 2015 (2 years and 6 months after LF + HS, MDO, 1 year and 11 months after GP) and December 13, 2013 (7 months after LF + HS, MDO, immediately after GP operation). A graph of SpO2, April 18, 2013, and October 10, 2013, and September 1, 2014.

**TABLE 2:** Timeline of date of first visit, date of cephalometric photography, date of PSG, date of surgery (LF + HS, MDO), date of surgery (GP)

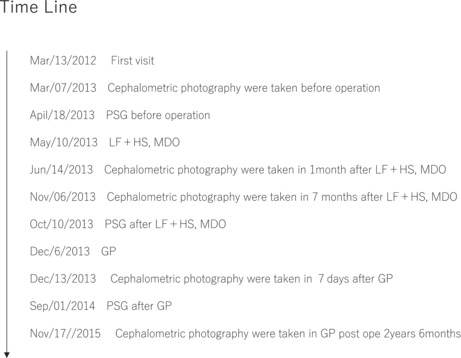

The moderate OSA diagnosed in the sleep outpatient department and the absence of obesity or palatine tonsil hypertrophy suggested that the morphological abnormality of the mandible, termed as small mandible, is the major cause of OSA.

Clinical diagnosis: micrognathia, malocclusion, open bite, OSA, and progressive condylar resorption (PCR).

Treatment plan: In this patient, the gingival exposure of the maxillary anterior teeth was as large as 5 mm, and the mandibular angle was large, and a long face was observed due to occlusal insufficiency due to small mandibular disease and open bite. Hence, the purpose was to improve the occlusal insufficiency and aesthetics. The patient received LF + HS on the maxilla, the palate and dentition were divided into horseshoe shapes, and the maxillary gingival fragments were planned to be elevated by 5 mm in the maxillary molars, 7 mm in the anterior teeth, and counterclockwise. In this case, the goal was to improve the OSA as well as the esthetic aspect and occlusion, but currently, there are no clear criteria regarding the amount of movement and improvement of OSA. Therefore, we explained to the patient that surgery may not improve OSA.

**FIGURE 4:**
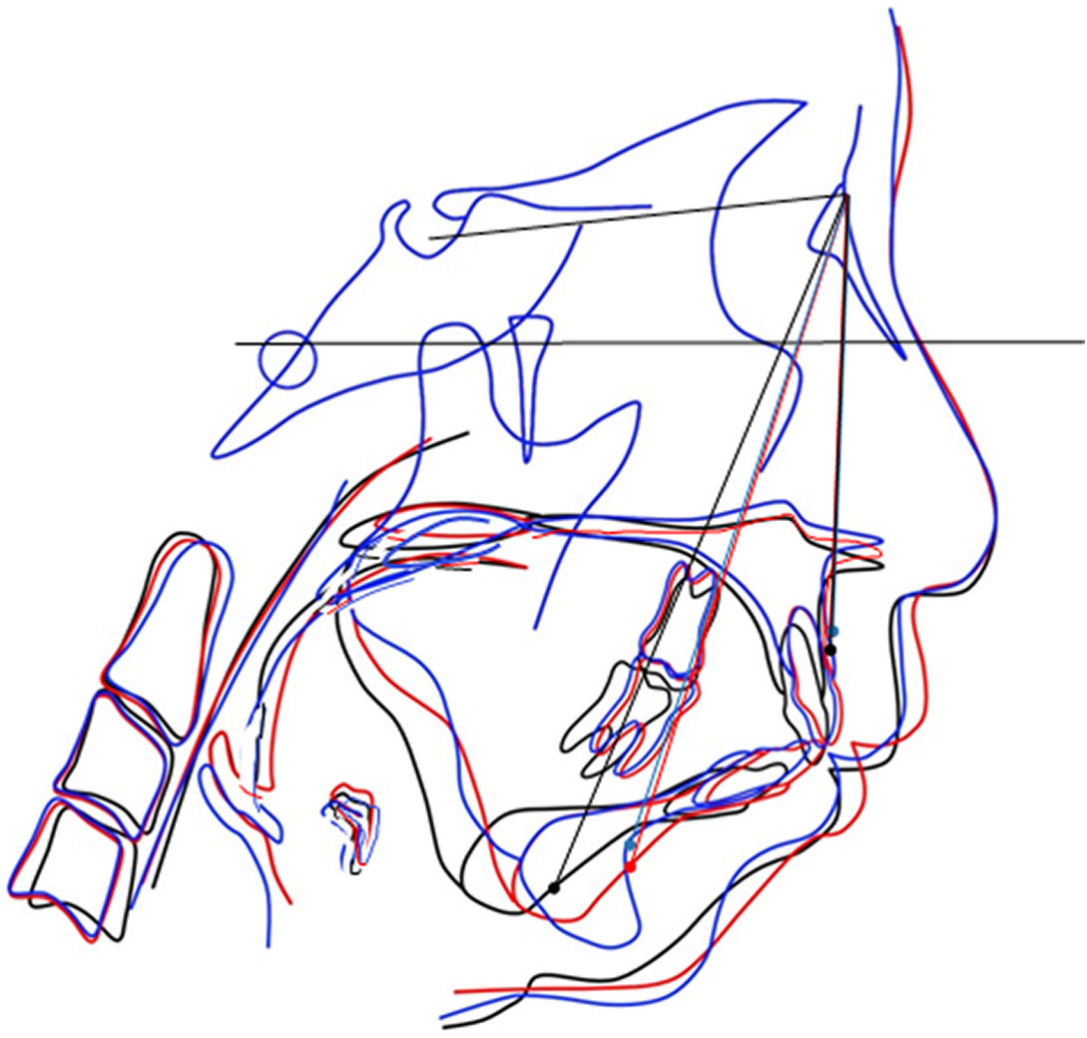
Superposition of cephalograms. Black line: Preoperative (March 7, 2013), Red line: LF + HS, 7 months after MDO (November 6, 2013), Blue line: LF + HS, 2 years and 6 months after MDO, 1 year and 11 months after GP (November 17, 2015).

Treatment and course: On May 10, 2013, both LF + HS and MDO were performed. Postoperative course: On October 10, 2013 (LF + HS, 5 months after MDO), the AHI decreased from 22.5 to 9.8/h, and the ESS score decreased from 14 to 2 points, and she said her drowsiness had disappeared (Tables 1 and 2). Moreover, in the cephalometric analysis of the cephalometric profile image obtained on November 6, 2013 (LF + HS, 7 months after MDO), the SNB was increased from 69° to 73° (Tables 1 and 2, Figures 3B and 4).

Intraoperative findings of GP: The small bone fragment was towed forward 13 mm and fixed with one Depuy Synthes Matrix LOCK Chin Plate.

Postoperative course: On September 1, 2014 (9 months after GP), the AHI further decreased from 9.8 to 7.8/h.

**FIGURE 5:**
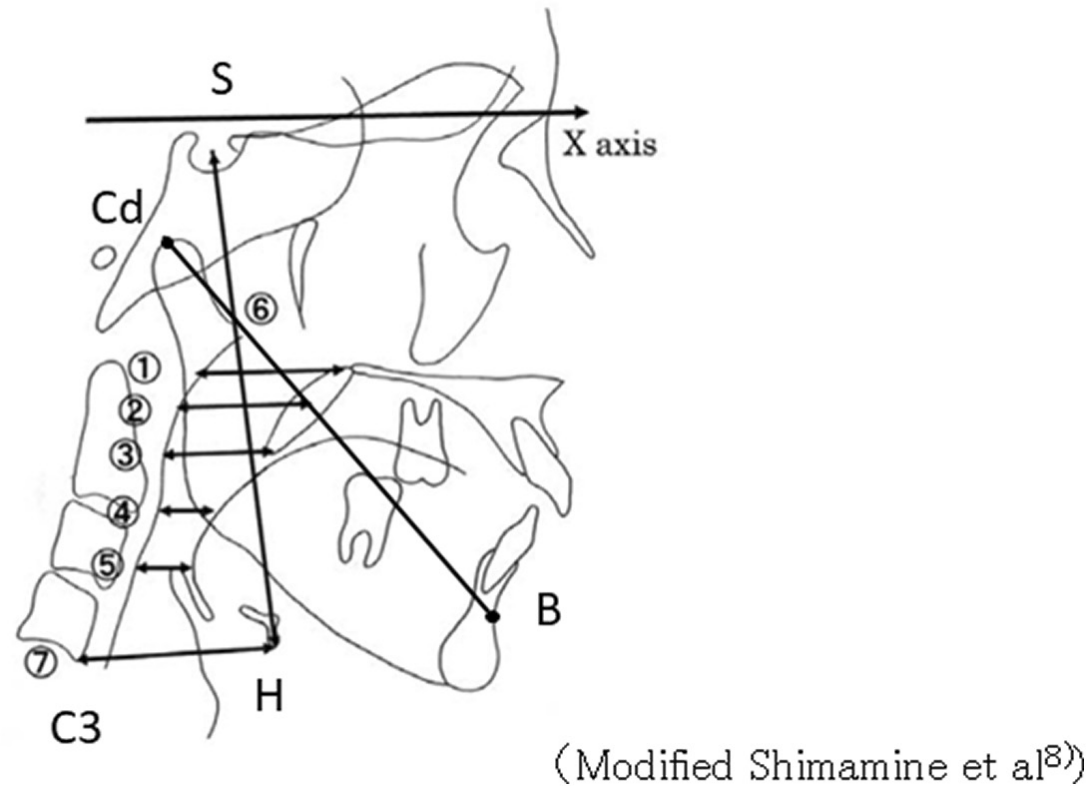
Measurement site (modified by Shimamine et al. 8)). Posterior soft palate: (1) PPS: Distance from the posterior pharyngeal wall to PNS. (2) SPPS: Distance from the posterior pharyngeal wall passing through the midpoint between the PNS and the lowest point of the soft palate to the soft palate. (3) MPS: Distance from the posterior wall of the pharynx passing through the lowest point of the soft palate to the soft palate. Posterior tongue: (4) IPS: Distance from the posterior pharyngeal wall through the lowest point of the anterior part of the second cervical spine to the tongue. (5) EPS: Distance from the posterior wall of the pharynx through the anterior end of the epiglottis to the tongue. These were measured parallel to the virtual FH plane. Hyoid bone position: (6) S‐H: Distance from the lowest point of the hyoid bone to Sella. (7) C3‐H: Distance from the lowest point of the hyoid bone to the lowest point of the anterior part of the third cervical spine. Mandibular body distance: Cd–B: Distance from Condylion to point B.

The anterior–posterior diameter of the pharynx: According to the method described by Shimamine et al., the anterior–posterior pharyngeal diameter and the amount of movement of the hyoid bone were measured using the cephalometric profile images taken before surgery, after LF + HS and MDO and GP (Figure 5, Tables 1 and 2).^5^

Regarding relapse: The amount of anterior extension of the mandible achieved by LF + HS and MDO was evaluated by measuring the distance between Condylion‐B points on both sides using the cephalometric profile image obtained before and after the modification of reported by Miyamoto et al. The amount of relapse was then calculated (Figure 5).^6^.

## 3 DISCUSSION AND CONCLUSIONS

Morphological changes in the upper respiratory tract and pharynx occur due to morphological changes and position movements of the mandible due to correction surgery, and changes in respiratory physiology are observed.^7^ Therefore, in Europe and the United States, one of the treatment methods for OSA is corrective surgery such as simultaneous upper and lower jawbone movement and genioglossus/anterior traction of the tongue muscle group, and its usefulness has also been reported^8^; however, it is not yet common in Japan. In our literature search, we found that LF + HS and MDO and GP were performed on patients with mandibular condyle who had resorption changes in the mandibular condyle, as in the present case, and had OSA.

Regarding the selection of technique: Regarding the maxillary procedure, LF has a limited amount of upward movement of the maxilla, but when HS is used in combination with LF, it is possible to move the maxillary bone fragment significantly upward. As it has been reported that LF + HS is indicated for cases with an upward movement of ≥4 mm,^9^ in the present case, the maxillary alveolar bone fragment was used for maxillary molars measuring 5 mm and anterior teeth measuring 7 mm.

Regarding the mandibular procedure, PCR is one of the complications after anterior mandibular movement by Sagittal Split Ramus Osteotomy (SSRO), and its incidence has been reported to be from 4.5% to 21%.^10^, ^11^ Strijen et al. mentioned that the one‐time anterior movement of the mandible by SSRO causes heavy burden on the mandibular condyle, and as a result of slow anterior extension of the mandible by MDO in 40 patients with PCR, we have reported the usefulness of MDO for these patients, describing that PCR was not observed in 39 of those patients (97.5%).^12^ Therefore, in the present case, in which an absorption image was observed in the mandibular condyle before surgery, MDO was selected to prevent the enhancement of PCR.

PCR: The distance from the mandibular condyle to the mandibular notch was measured using 3D‐CT images obtained preoperatively (March 7, 2013) and 1 year and 3 months after LF + HS and MDO (August 14, 2014). Comparing the distances, the right side decreased by 0.4 mm from 12.5 to 12.1 mm, and the left side decreased by 1.6 mm from 8.3 to 6.7 mm. In other words, the right side was almost the same as before the operation, but the left side demonstrated mild bone resorption.

Relapse: Regarding relapse, McCarthy et al. reported a rate of 22% and Miyamoto et al. reported a rate of 30%–40% relapse.^6^, ^13^ In the present case, a difference of +11.8 mm on the right side and +9.1 mm on the left side was observed between before surgery and immediately after LF + HS and MDO and 8.7 mm on the right side compared with values measured 2 years and 6 months after LF + HS and MDO surgery. The left side was 6.1 mm, the right side was 25.6% relapse, and the left side was 33.0% relapse (Tables 1 and 2, Figures 1C,D).

The anterior–posterior diameter of the pharynx: Comparing the posterior part of the soft palate before surgery with that 7 months after LF + HS and MDO (November 6, 2013), (1) PPS decreased from 35.1 to 34.8 mm (−0.3 mm), and (2) SPPS increased from 17.7 to 19.3 mm (+1.6 mm), and (3) MPS increased from 4.6 to 10 mm (+5.4 mm).

In the posterior part of the tongue, (4) IPS increased from 7.4 to 11.2 mm (+3.8 mm), and (5) EPS increased from 6.7 to 10.3 mm (+3.6 mm). It is considered that this finding is because the tongue tuft became larger due to the anterior extension of the mandible by MDO, and the anterior–posterior diameter of the pharynx behind the tongue increased due to the anterior extension of the tongue.

Regarding the position of the hyoid bone: Comparing preoperative values and those measured 7 months after LF + HS and MDO (November 6, 2013), it was observed that (6) S‐H decreased from 106.4 to 104.4 mm (−2.0 mm), and (7) C3‐H decreased from 42.7 to 48.2. It increased to mm (+5.5 mm), and the hyoid bone moved upward and anteriorly. It is speculated that this is because the hyoid muscles and geniohyoid muscles attached to the hyoid bone and geniohyoid spines were pulled upward and anteriorly by the MDO, and the hyoid bones to which these muscles were attached moved upward and anteriorly.

Comparing the values measured immediately before GP (November 6, 2013) and after GP (December 13, 2013), it was observed that (4) IPS behind the tongue increased from 11.2 to 11.8 mm (+0.6 mm), and (5) EPS increased from 10.3 to 8.6. It decreased to (−1.7 mm).

Comparing the values of (6) S‐H and (7) C3‐H, which indicate the position of the hyoid bone, immediately before GP (December 13, 2013) and 1 week after GP (December 13, 2013), (6) S‐H was 104.4 mm. It increased from 107.8 mm (+3.4 mm), and (7) C3‐H decreased from 48.2 to 46.5 mm (−1.7 mm). Since GP pulls the genioglossus muscle and geniohyoid muscle attached to the genioglossus anteriorly, it is considered that the hyoid bone moves upward and anteriorly after GP. However, as indicated by Shimamine et al., MDO performed before GP caused the infrahyoid muscles that had once stretched to contract again as the anterior movement of the mandible caused the tongue to expand forward.^5^ Therefore, (5) EPS decreased, and it was considered that the force to move the hyoid bone downward was exerted.

The increase in pharyngeal anterior–posterior diameter before surgery, after LF + HS and MDO and after GP, was the largest in (1) PPS (+6.1 mm). This result and AHI, which was 22.5/h at the first visit, decreased significantly to 7.8/h after all surgeries (LF + HS and MDO and GP), and daytime sleepiness disappeared completely as a clinical symptom. Therefore, it was speculated that the site of obstruction in the present case was the posterior part of the soft palate, especially (1) PPS.

After LF + HS and MDO surgery, drowsiness did not appear during the daytime, and fortunately, in the present case, the corrective surgery was able to improve both small mandibular disease and OSA. However, the criteria for adaptation and the prediction of improvement in symptoms associated with jawbone movement are not yet sufficient. In this case, in order to improve OSA as well as the esthetic aspect and occlusion, a maxillary and mandibule osteotomy was performed to enlarge the hypopharynx and increase airflow. Osteotomy was performed to enlarge the upper, middle, and lower pharynx to increase airflow. As a result, OSA was improved. However, there is no clear standard for the relationship between the amount of jaw bone movement and airway ventilation, and it is necessary to study the relationship between the amount of jaw bone movement and improvement of OSA by accumulating more cases. In addition, Takehiro et al. report that it is necessary to consider the balance between the amount of change in soft tissues and the size of bone around the soft tissue for the mechanism of OSA onset^14^; also, it is necessary to consider that not only changes in bone movement but also neural regulatory mechanisms by orthognatic surgery are involved. Moreover, it is necessary to evaluate the size of soft tissues such as the tongue and soft palate and analyze the functional changes, and we believe that elucidation of these aspects will be the future task.

## AUTHOR CONTRIBUTIONS

Shinya Koshinuma and Takafumi Fujii involved in conception and design of study, acquisition of data, analysis and/or interpretation of data, and drafting the manuscript. Takeshi Okamura involved in acquisition of data. Yasuyuki Asada involved in analysis and/or interpretation of data. Yoshisato Machida involved in revising the manuscript critically for important intellectual content. Gaku Yamamoto involved in conception and design of study, and revising the manuscript critically for important intellectual content. All authors critically revised the report, commented on drafts of the manuscript, and approved the final report.

## CONFLICT OF INTEREST

There are no conflicts of interest to disclose regarding this paper.

## ETHICAL APPROVAL

Written informed consent was obtained from the patient to publish this case report, laboratory data, and accompanying clinical images. Any investigation on the patient was performed in accordance with the Declaration of Helsinki.

## CONSENT

Written informed consent was obtained from the patient to publish this case report, laboratory data, and accompanying clinical images.

## Data Availability

The data that support the findings of this study are available from the corresponding author upon request.
